# Myeloid GHSR Deficiency Protects Against Endotoxemia via Macrophage Mitochondrial Reprogramming

**DOI:** 10.3390/biomedicines14081668

**Published:** 2026-07-24

**Authors:** Da Mi Kim, Zheng Shen, Quan Pan, Zeyu Liu, Wanbao Yang, Natividad R. Fuentes, Robert S. Chapkin, Gus A. Wright, Bhimanagouda Patil, Shaodong Guo, Yuxiang Sun

**Affiliations:** 1 Department of Nutrition, Texas A&M University, College Station, TX 77843, USA; dmkim5322@tamu.edu (D.M.K.); zheng.shen@ag.tamu.edu (Z.S.); quan.pan@ag.tamu.edu (Q.P.); lzyciacustom@tamu.edu (Z.L.); wanbao.yang@ag.tamu.edu (W.Y.); nrfuentes@mdanderson.org (N.R.F.); robert.chapkin@ag.tamu.edu (R.S.C.); shaodong.guo@ag.tamu.edu (S.G.); 2 Department of Cancer Biology, University of Texas MD Anderson Cancer Center, Houston, TX 77030, USA; 3 Department of Veterinary Pathobiology, Texas A&M University, College Station, TX 77843, USA; gwright@cvm.tamu.edu; 4 Vegetable and Fruit Improvement Center, Department of Horticultural Sciences, Texas A&M University, College Station, TX 77845, USA; bhimanagouda.patil@ag.tamu.edu

**Keywords:** macrophage, growth hormone secretagogue receptor (GHSR), endotoxemia, lipopolysaccharide (LPS), mitochondrial function, reactive oxygen species (ROS)

## Abstract

**Background**: Endotoxemia is a severe inflammatory condition that is characterized by acute immune responses and oxidative stress; endotoxemia can further develop into a cytokine storm and sepsis leading to severe organ damage. Our recent studies revealed that the growth hormone secretagogue receptor (GHSR) regulates macrophage polarization in obesity- and aging-associated chronic inflammation. However, its role in acute inflammation during endotoxemia remains unclear. **Methods**: We subjected myeloid-specific *Ghsr* knockout mice (*LysM-Cre*;*Ghsr*^*f/f*^) to lipopolysaccharide (LPS)-induced endotoxemia *in vivo* and treated bone marrow-derived macrophages (BMDMs) with LPS *in vitro*. Subsequently, mouse survival rate and inflammatory signatures in the blood, peritoneal cavity, liver, and BMDM were assessed. In the *ex vivo* study, conditioned medium (CM) from BMDMs was applied to primary hepatocytes to assess how BMDM-derived CM influences hepatocyte inflammatory responses. **Results**: Myeloid-specific *Ghsr* knockout mice exhibited a significantly improved survival rate following LPS-induced endotoxemia, accompanied by reduced systemic inflammation, evident in the blood, peritoneal macrophages, and liver. In addition, *Ghsr* deficiency suppressed LPS-induced caspase-1 activation and pro-inflammatory cytokine secretion in macrophages. Consistent with these results, conditioned media from *Ghsr*-deficient BMDMs attenuated the inflammatory responses of primary hepatocytes. Mechanistically, LPS increased GHSR expression in BMDMs, and *Ghsr*-deficient BMDMs activated mitochondrial respiration and suppressed production of mitochondrial reactive oxygen species (ROS), resulting in downregulation of inflammatory activation of macrophages following LPS exposure. **Conclusions**: These data demonstrate that macrophage GHSR promotes systemic and tissue inflammation during endotoxemia by regulating mitochondria-associated macrophage polarization. The findings suggest that macrophage GHSR may represent a promising immunomodulatory target for acute inflammatory states, including endotoxemia and sepsis.

## 1. Introduction

Endotoxemia, characterized by the presence of circulating endotoxins—primarily lipopolysaccharide (LPS) derived from gram-negative bacteria—is a major driver of systemic inflammation in gram-negative sepsis [1,2]. Excessive endotoxin exposure can provoke dysregulated cytokine production, multiple-organ dysfunction, and mortality [3,4,5,6]. Although the precise pathogenesis of endotoxemia remains incompletely understood, dysregulated innate immune responses, particularly those involving macrophages, contribute substantially to disease initiation and progression and to sepsis-associated organ dysfunction [7,8]. Macrophages are highly plastic innate immune cells that contribute to tissue homeostasis and coordinate host defense, inflammatory, and tissue-repair responses across diverse physiological and pathological conditions [9,10,11]. Accordingly, cues within the tissue microenvironment can drive macrophages toward distinct polarization states with divergent functional consequences [11,12,13].

Macrophage polarization has traditionally been broadly categorized into classically activated, pro-inflammatory M1-like and alternatively activated, anti-inflammatory or tissue-reparative M2-like states based on their inducing stimuli, molecular signatures, and cytokine profiles. Classically activated M1-like macrophages induced by LPS and/or interferon-gamma generally exhibit enhanced glycolysis, impaired mitochondrial oxidative phosphorylation (OXPHOS), and elevated pro-inflammatory cytokine production [14,15,16,17]. Compared with M1-like macrophages, interleukin (IL)-4- and/or IL-13-induced M2-like macrophages generally rely more heavily on mitochondrial oxidative metabolism and display a less pro-inflammatory cytokine production, although glycolysis may also contribute to their activation [18,19,20,21]. M1-like macrophage activation is associated with glycolytic overdrive and mitochondrial dysfunction, that are key features of endotoxemia [22,23].

Pro-inflammatory M1-like macrophages rely on glycolysis, whereas anti-inflammatory M2-like macrophages rely more heavily on OXPHOS to meet their energy demands [24,25]. Beyond this glycolytic shift, mitochondrial function also contributes substantially to M1-like macrophage activation. During M1 polarization, pyruvate utilization and mitochondrial metabolism are reprogrammed, accompanied by restructuring of the TCA cycle and accumulation of metabolic intermediates that further modulate mitochondrial function and inflammatory signaling [21,24,26,27]. In pro-inflammatory M1-like macrophages, mitochondria contribute to inflammatory signaling in part through the generation of mitochondrial reactive oxygen species (mtROS) [28,29,30]. Elevated mitochondrial oxidative stress can further activate major inflammatory pathways, including nuclear factor-κB (NF-κB) signaling [31,32]. In addition, excessive mtROS production under LPS-induced inflammatory conditions can promote aberrant activation of other important inflammatory pathways, including inflammasome-associated caspase-1 activation [33,34]. LPS-induced Toll-like receptor 4 signaling has also been linked to mitochondrial OXPHOS dysfunction and a metabolic shift from OXPHOS toward glycolysis in activated macrophages [30,35,36,37]. Therefore, metabolic reprogramming is an important determinant of macrophage activation and inflammatory function and may contribute to the pathological progression of endotoxemia.

Growth hormone secretagogue receptor (GHSR), a G protein-coupled receptor (GPCR) featuring the typical seven transmembrane domains, is the only known receptor for the nutrient-sensing gut hormone ghrelin [38]. GPCRs are favorable drug targets, as over 50% of drugs on the market target this unique type of receptors [39]. Ghrelin–GHSR signaling is classically recognized for its roles in appetite stimulation, body-weight gain, and the regulation of glucose and lipid metabolism [40]. We have shown that GHSR alters mitochondrial function in pancreatic islets and brown adipose tissues [41,42,43]. Our recent studies further revealed that GHSR is an important driver of inflammation for both adipose tissue and liver under metabolically stressed conditions, such as obesity and aging [43,44,45,46]. However, current literature on ghrelin-GHSR signaling in inflammation have been primarily focused on chronic or metabolic inflammatory settings, while its roles in acute and severe inflammatory conditions, such as endotoxemia and sepsis, remain poorly understood. Some evidence suggests that ghrelin exerts context-dependent effects during sepsis, displaying a protective effect in the early stage but an adverse effect at the late stage of inflammation [47]. Nevertheless, the cellular and metabolic mechanisms underlying these stage-specific effects, particularly in innate immune cells, have not been elucidated. In addition, it has been shown that ghrelin has both GHSR-dependent and GHSR-independent effects [40,48,49,50]. GHSR, as a GPCR, is known to have high constitutive activity and can be activated even in the absence of ghrelin [51,52,53,54]. The unique property of GHSR likely contributes to the inconsistent findings reported in the literature regarding ghrelin and GHSR.

Macrophages play distinct and context-dependent roles in acute versus chronic inflammation [55,56]. During acute inflammatory responses such as endotoxemia and sepsis, macrophages rapidly undergo functional and metabolic reprogramming to initiate innate immune defenses [57,58]. In contrast, sustained macrophage activation under chronic inflammatory states promotes persistent inflammation and tissue damage [55,59,60]. GHSR signaling has been implicated in metabolic regulation and chronic inflammatory conditions, but its cell-autonomous role in macrophages during acute endotoxemia, particularly with respect to mitochondrial function and inflammatory reprogramming, remains largely unexplored. In the present study, we investigated the effects of macrophage *Ghsr* deficiency in LPS-induced endotoxemia and demonstrated that GHSR functions as a key regulator of endotoxemia by modulating macrophage mitochondrial function to promote pro-inflammatory reprogramming.

## 2. Materials and Methods

### 2.1. Animal Experiments

Previously we reported the generation of fully backcrossed *Ghsr*-floxed mice in the C57BL/6J background [61]. Using a Cre-Lox system, we generated myeloid-specific *Ghsr*-deficient (*LysM-Cre;Ghsr^f/f^*) mice by breeding *Ghsr^f/f^* mice with myeloid-specific *LysM-Cre* [46,62]. Mice were housed in the animal facility of Texas A&M University (College Station, TX, USA), maintained on 12 h light and 12 h dark cycles (lights on at 6:00 AM) at 75 °F ± 1 °F. Food and water were available ad libitum.

### 2.2. Endotoxin-Induced Endotoxemia Model

Endotoxemia was induced in age-matched 3-month-old male *Ghsr^f/f^* (control) and *LysM-Cre;Ghsr^f/f^* (myeloid-specific *Ghsr*-deficient) mice by single *i.p.* injection of bacterial endotoxin LPS (*Escherichia coli* O55:B5; Sigma-Aldrich, St. Louis, MO, USA) at 5 mg/kg body weight (BW) [63,64,65], and survival was recorded daily. Blood, peritoneal macrophages, and liver tissue were collected 24 h following LPS administration. All experiments were conducted following the NIH guidelines and approved by the institutional animal care and use committee.

### 2.3. BMDM Isolation and Culture

Bone marrow cells were cultured from control and *LysM-Cre;Ghsr^f/f^* mice as previously described [46,66]. In brief, cells were differentiated on 6-well plates at a density of 1.5 × 10^6^ cells per well in a humidified incubator at 37 °C with 5% CO_2_ for 7 days. At the end of the 7-day culture period, BMDMs were treated with LPS (100 ng/mL). The conditioned medium was collected to measure cytokine levels.

### 2.4. Primary Hepatocyte Isolation and Co-Culture with Conditioned Medium (CM) from BMDMs

Primary mouse hepatocytes were isolated using a two-step collagenase perfusion method [67,68]. Briefly, primary hepatocytes were isolated from 3-month-old control mice and cultured overnight in DMEM with 10% FBS, 1% penicillin-streptomycin (P/S). CM of BMDMs (1.5 × 10^6^ cells per well in 6-well plates) was collected after 24 h of LPS treatment, then applied to primary hepatocytes for an additional 24 h for co-culture experiments as previously described [46,69,70]. The expression of pro-inflammatory genes in hepatocytes was subsequently studied by real-time PCR.

### 2.5. Peritoneal Macrophage (PM) Isolation

PMs were obtained from the peritoneum of *Ghsr^f/f^* or *LysM-Cre;Ghsr^f/f^* mice 24 h following *i.p.* injection of a single dose of LPS at 5 mg/kg BW [71]. Briefly, after anesthetization with isoflurane, mice were euthanized by rapid cervical dislocation and 3 mL of ice-cooled PBS with 2% fetal bovine serum was injected into the abdominal cavity. After gentle shaking for 3 min, abdominal fluid was extracted using an 18G needle. Red blood cells were lysed with ACK lysis buffer for 5 min, and the reaction was quenched by adding two times the volume of PBS. PMs were then collected by centrifugation at 300× *g* for 5 min. The PMs were subjected to RNA isolation (using 1.5 × 10^6^ cells) and flow cytometry analysis (using 2 × 10^5^ cells).

### 2.6. Flow Cytometry Analysis

Collected PMs were washed twice with PBS and stained with live/dead aqua (Thermo Fisher Scientific, Waltham, MA, USA) for 30 min on ice. Cells were then washed with FACS buffer and incubated with anti-FcγR II/III antibody (BD Biosciences, San Jose, CA, USA) for 15 min. PMs were stained for 30 min on ice with a mixture of fluorescently labeled antibodies against surface markers including APC-cy7 anti-mouse CD11b antigen, a cell surface marker for myeloid cells (BioLegend, San Diego, CA, USA), and PE-cy7 anti-mouse F4/80 antigen, a cell surface marker for mature mouse macrophages (eBioscience, San Diego, CA, USA). PMs were then further washed and permeabilized for 30 min on ice. To assess intracellular markers such as TNFα, IL1β and IL6, PMs were stained for 30 min on ice with PE-Dazzle594 anti-mouse TNFα antigen (Biolegend, San Diego, CA, USA), PE anti-mouse IL1β antigen (Thermo Fisher Scientific, Waltham, MA, USA), APC anti-mouse IL6 antigen (BD Biosciences, San Jose, CA, USA), and Alexa Fluor488 anti-mouse iNOS antigen (Thermo Fisher Scientific, Waltham, MA, USA), respectively. Finally, cells were washed before data acquisition with MoFlo Astrios EQ (Beckman Coulter Life Sciences, Indianapolis, IN, USA) and analyzed using FlowJo software, version 10.10.0 (Tree Star, Inc., Ashland, OR, USA).

### 2.7. Quantitative Real-Time PCR

Total RNA was isolated using TRIzol Reagent (Invitrogen, Carlsbad, CA, USA) or Aurum Total RNA mini kit (Bio-Rad Laboratories, Inc., Hercules, CA, USA) according to the manufacturer’s instructions [72]. The mRNA samples were treated with RNase-free DNase (Ambion, Austin, TX, USA) to remove genomic DNA before reverse transcription. Reverse transcription was performed using Superscript III First Strand Synthesis System (Invitrogen, Carlsbad, CA, USA) according to the manufacturer’s instructions. Quantitative real-time PCR reaction was performed as we previously described [73]. Relative gene expression levels were calculated using the 2^−∆∆Ct^ method [74]. *Ghsr-1a* primers were designed to flank the intron as follows: sense primer 5′-GGACCAGAACCACAAACAGACA-3′, anti-sense primer 5′-CAGCAGAGGATGAAAGCAAACA-3′ [75]. Additional primer information is available upon request.

### 2.8. Western Blotting

BMDMs (3 × 10^6^ per well in 6-well cell culture plates) were treated with LPS (100 ng/mL) and washed with DPBS before making the cell lysate. Proteins were prepared from BMDMs using RIPA buffer with protease and phosphatase inhibitor (Roche Diagnostics GmbH, Mannheim, Germany), resolved by SDS-PAGE, and transferred to PVDF membrane for immunoblotting analysis using antibodies such as GHSR (Thermo Fisher Scientific, cat# 720278, Waltham, MA, USA) and GAPDH (Cell Signaling Technology, cat# 2118, Danvers, MA, USA) [54]. The intensity of the protein signal was analyzed by Image J software, version 1.54g.

### 2.9. Cytokine Analysis

For cytokine analysis, blood was collected by retro-orbital bleeding under anesthesia and allowed to clot at room temperature. Following centrifugation (13,000 rpm, 4 °C, 15 min), serum was collected and stored at −80 °C until assessment for cytokine levels. CM from BMDMs (1.5 × 10^6^ cells/well in 6-well plates) was collected 24 h after LPS treatment. Cytokine levels were analyzed using the Millipore multiplex assay (Milliplex Map kit, cat# MHSTCMAG-70K; EMD Millipore Corporation, Billerica, MA, USA) and Luminex reader according to the manufacturer’s instructions.

### 2.10. Caspase-1 Activity

At 4 h after LPS treatment, BMDMs were lysed with cell lysis buffer provided in the Caspase-1 Fluorometric Assay Kit (Abcam, cat# ab39412, Cambridge, UK), which is based on the cleavage of the substrate YVAD-AFC (AFC: 7-amino-4-trifluoromethyl coumarin) and the quantification of free AFC (excitation/emission: 400/505 nm). After a 2 h incubation in reaction buffer containing the YVAD-AFC substrate, fluorescence intensity was measured using a fluorescent microplate reader.

### 2.11. Seahorse Analysis of Mitochondrial Oxidative Phosphorylation

An XF96 analyzer (Seahorse Bioscience, Inc., North Billerica, MA, USA) was used to measure functional bioenergetics of intact BMDMs in real-time. BMDMs were seeded into Seahorse Bioscience XF 96 cell culture plates at the seeding density of 50,000 cells in 200 µL medium and allowed to adhere and grow for 24 h in a 37 °C humidified incubator with 5% CO_2_. Measurements of extracellular flux were made in bicarbonate-free Seahorse XF assay medium. Oxygen consumption rate (OCR) was calculated at baseline to compare the rate of OXPHOS. Mitochondrial function was analyzed by sequentially adding the pharmacological inhibitors of OXPHOS. The number of cells in each well was measured using CyQUANT Cell Proliferation Assay Kit (Invitrogen) to normalize the data. The results were analyzed using XFe Wave software, version 2.6.4.24 (Seahorse Bioscience).

### 2.12. Measurement of Mitochondrial Superoxide

Mitochondrial superoxide was determined using MitoSOX Red and MitoTracker Green. BMDMs were cultured in 35 mm round dish at a density of 800,000 cells per dish. At 1 h of LPS treatment, the medium was changed, nuclear staining dye Hoechst (final concentration 1 µg/mL), MitoTracker Green (final concentration 175 nM) and MitoSOX Red (final concentration 2.5 µM) were added. After the staining, cells were washed and suspended in live cell solution. Fluorescence was then acquired at excitation/emission wavelengths of 405/430–480 nm for Hoechst, 488/500–550 nm for MitoTracker Green, and 561/600–700 nm for MitoSOX Red. The mitochondrial superoxide data were normalized to the nuclear stain and quantified.

### 2.13. Statistical Analysis

Statistical analyses were performed with GraphPad Prism 8.01. Graphical data are presented as means ± SEM. Statistical analyses were performed to compare two groups using a two-tailed Student’s *t*-test, and to compare three or more groups using either one-way or two-way ANOVA followed by Tukey’s post hoc test. * (or #) *p* < 0.05, ** (or ##) *p* < 0.01, and *** (or ###) *p* < 0.001 were considered statistically significant.

## 3. Results

### 3.1. LPS Increases GHSR Expression in Macrophages, and Myeloid-Specific Ghsr Deficiency Suppresses Cytokine Levels and Reduces Mortality

**Figure 1:**
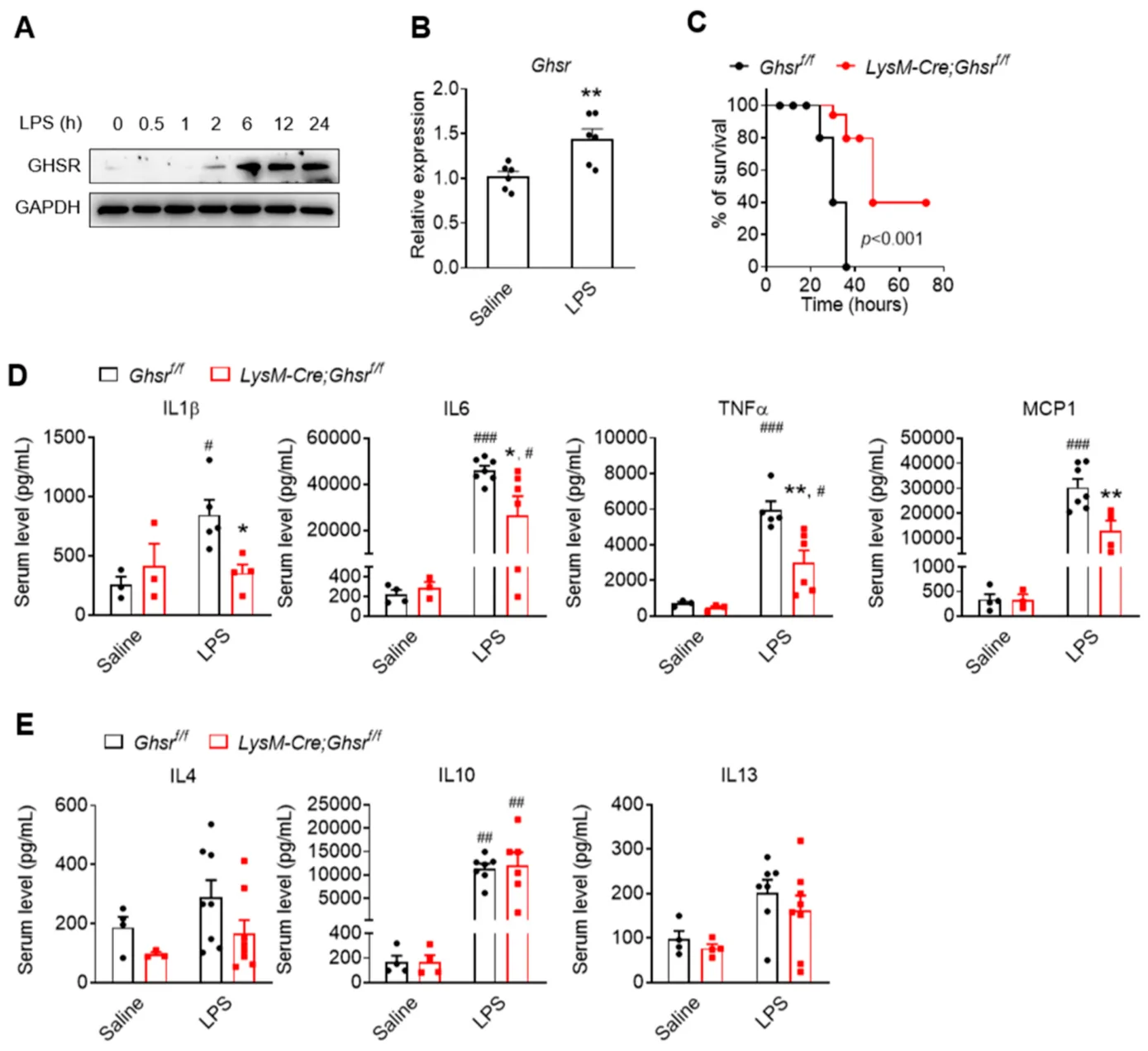
LPS increases GHSR expression in macrophages, and myeloid-specific *Ghsr* deficiency attenuates systemic inflammation in LPS-induced endotoxemia. BMDMs were treated with 100 ng/mL of LPS. Mice were intraperitoneally (*i.p.*) injected with saline or LPS (5 mg/kg BW). Blood was collected 24 h after saline or 5 mg/kg BW LPS *i.p.* injection. (**A**) Representative Western blot image of GHSR protein levels in LPS-treated BMDMs. (**B**) Gene expression of *Ghsr* in BMDMs treated with LPS for 4 h, n = 6/group. (**C**) Survival rate of control (*Ghsr^f/f^*) and *LysM-Cre;Ghsr^f/f^* mice in LPS-induced endotoxemia (n = 13–17 mice/group). (**D**,**E**) Serum levels of pro-inflammatory (**D**) and anti-inflammatory (**E**) cytokines (n = 3–8 mice/group). Data are presented as mean ± SEM. * *p* < 0.05, ** *p* < 0.01, *LysM-Cre;Ghsr^f/f^* vs. *Ghsr^f/f^*; # *p* < 0.05, ## *p* < 0.01, and ### *p* < 0.001, LPS vs. saline.

In BMDM culture, GHSR expression at the protein level was significantly increased by LPS treatment (Figure 1A), *Ghsr* mRNA expression was also significantly upregulated (Figure 1B), although the magnitude of change was smaller than that observed at the protein level, likely due to the temporal difference between transcription and translation. The increases in GHSR protein and *Ghsr* gene expression suggest that *Ghsr* is transcriptionally responsive to endotoxic insult. Since *i.p.* injection of LPS at a dose of 5 mg/kg body weight (BW) has been established in the literature as a regimen that can induce severe endotoxemia in mice [64,76,77], we examined the effect of macrophage GHSR on the LPS-induced acute inflammation of endotoxemia *in vivo*. Myeloid-specific *Ghsr*-deficient mice (*LysM-Cre;Ghsr^f/f^* mice) and controls (*Ghsr^f/f^* mice) were treated with 5 mg/kg BW LPS *i.p.*, and survival rate was assessed. Importantly, macrophage *Ghsr* deficient mice showed improved survival following LPS-induced endotoxemia, resulting in improved survival (Figure 1C). This finding suggests that myeloid-specific *Ghsr* deficiency reduces endotoxemia-associated mortality.

Consistent with these observations, myeloid-specific *Ghsr* deficiency significantly reduced LPS-induced pro-inflammatory cytokine levels (IL1β, IL6, TNFα, MCP1) in the serum of the mice by 53.0%, 42.7%, 49.5%, and 57.2%, respectively (Figure 1D), while the anti-inflammatory cytokines (IL4, IL10, IL-13) were comparable (Figure 1E). These findings indicate that macrophage GHSR plays an important role in the regulation of systemic cytokines, primarily affecting pro-inflammatory activation.

### 3.2. Myeloid-Specific Ghsr Deletion Attenuates Pro-Inflammatory Responses of PMs

**Figure 2:**
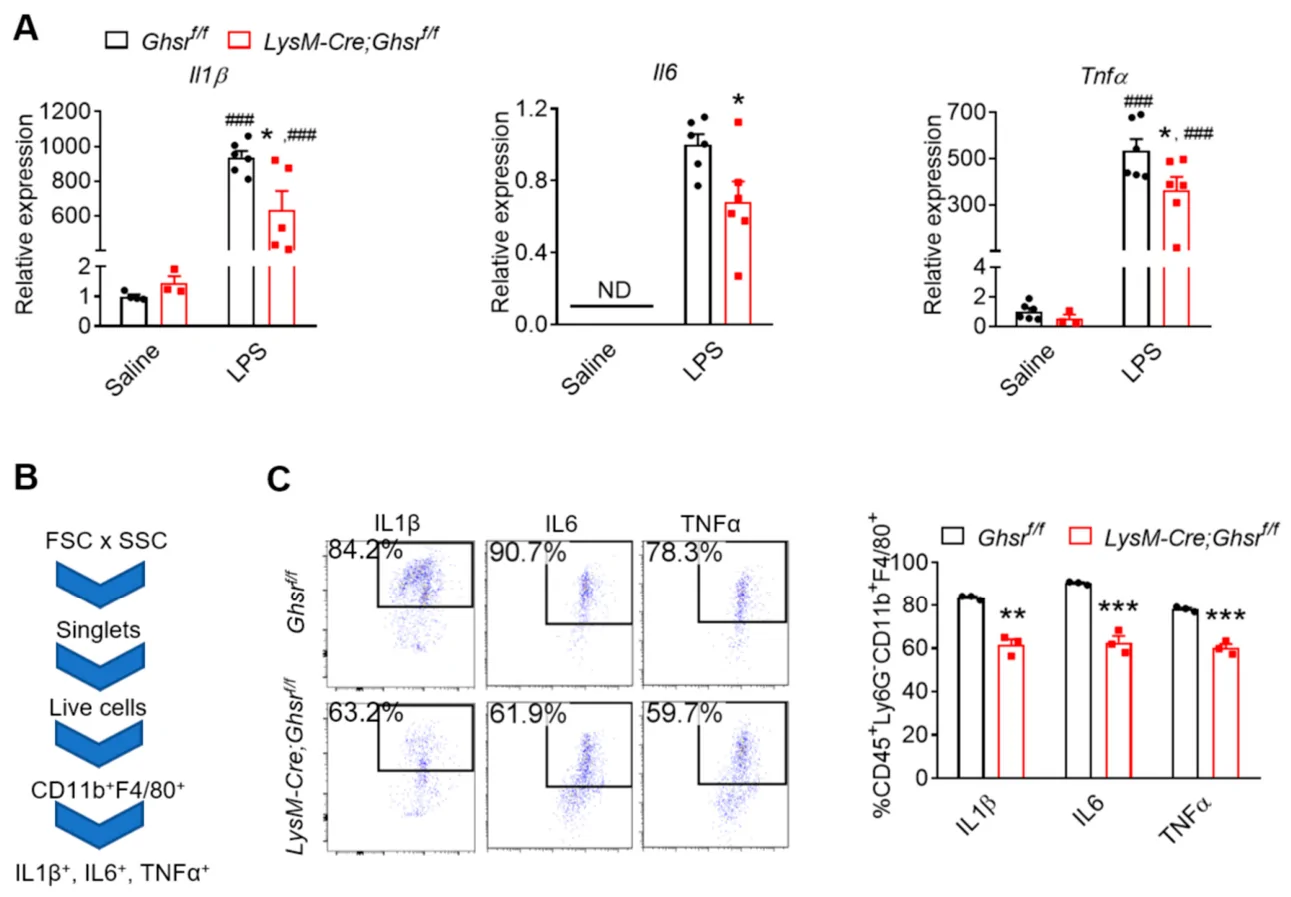
Myeloid-specific *Ghsr* deficiency attenuates inflammatory activation of PMs following LPS-induced endotoxemia. PMs from mice were collected 24 h following saline or 5 mg/kg BW LPS *i.p.* injection. (**A**) mRNA level of pro-inflammatory cytokine-related genes in PMs of control and *LysM-Cre;Ghsr^f/f^* mice (n = 3–6 mice/group). (**B**) Flow cytometry gating strategies of PMs. (**C**) Percentage of pro-inflammatory cytokine subsets in PMs from control and *LysM-Cre;Ghsr^f/f^* mice following LPS injection (n = 3 mice/group). Data are presented as mean ± SEM. * *p* < 0.05, ** *p* < 0.01, *** *p* < 0.001, *LysM-Cre;Ghsr^f/f^* vs. *Ghsr^f/f^*; ### *p* < 0.001, LPS vs. saline; ND, not detected.

To further validate the role of macrophage GHSR in endotoxemia *in vivo*, PMs were obtained from the peritoneum 24 h after LPS administration (5 mg/kg BW). As expected, qPCR analysis revealed that the mRNA expression of *Il1β*, *Il6*, and *Tnfα* was significantly upregulated in PMs from control mice (Figure 2A). Remarkably, macrophage *Ghsr* deficiency decreased LPS-induced *Il1β*, *Il6*, and *Tnfα* mRNA levels in PMs by 32.5%, 31.9%, and 32.0%, respectively (Figure 2A). Furthermore, we performed flow cytometry analysis of PMs using the gating strategies shown in Figure 2B. Since the LysM promoter induces Cre activity in myeloid cells, we used CD11b to gate myeloid cells in peritoneal fluid and further gate F4/80 to distinguish mature macrophages to examine the effect of GHSR on macrophages. Intracellular markers such as IL1β, IL6, and TNFα were gated in the population of macrophages labeled with CD11b and F4/80 (CD11b^+^F4/80^+^) fluorescence-tagged antibodies. Myeloid *Ghsr* deficiency significantly reduced intrinsic expression of IL1β by 26.4%, IL6 by 30.6%, and TNFα by 23.0% (Figure 2C). These results indicate that macrophage GHSR alters the inflammatory response *in vivo* under acute inflammation, regulating pro-inflammatory cytokine expression in the macrophages.

### 3.3. Myeloid-Specific Ghsr Deficiency Attenuates LPS-Induced Pro-Inflammatory Cytokine Secretion and Caspase-1 Activity in BMDMs

**Figure 3:**
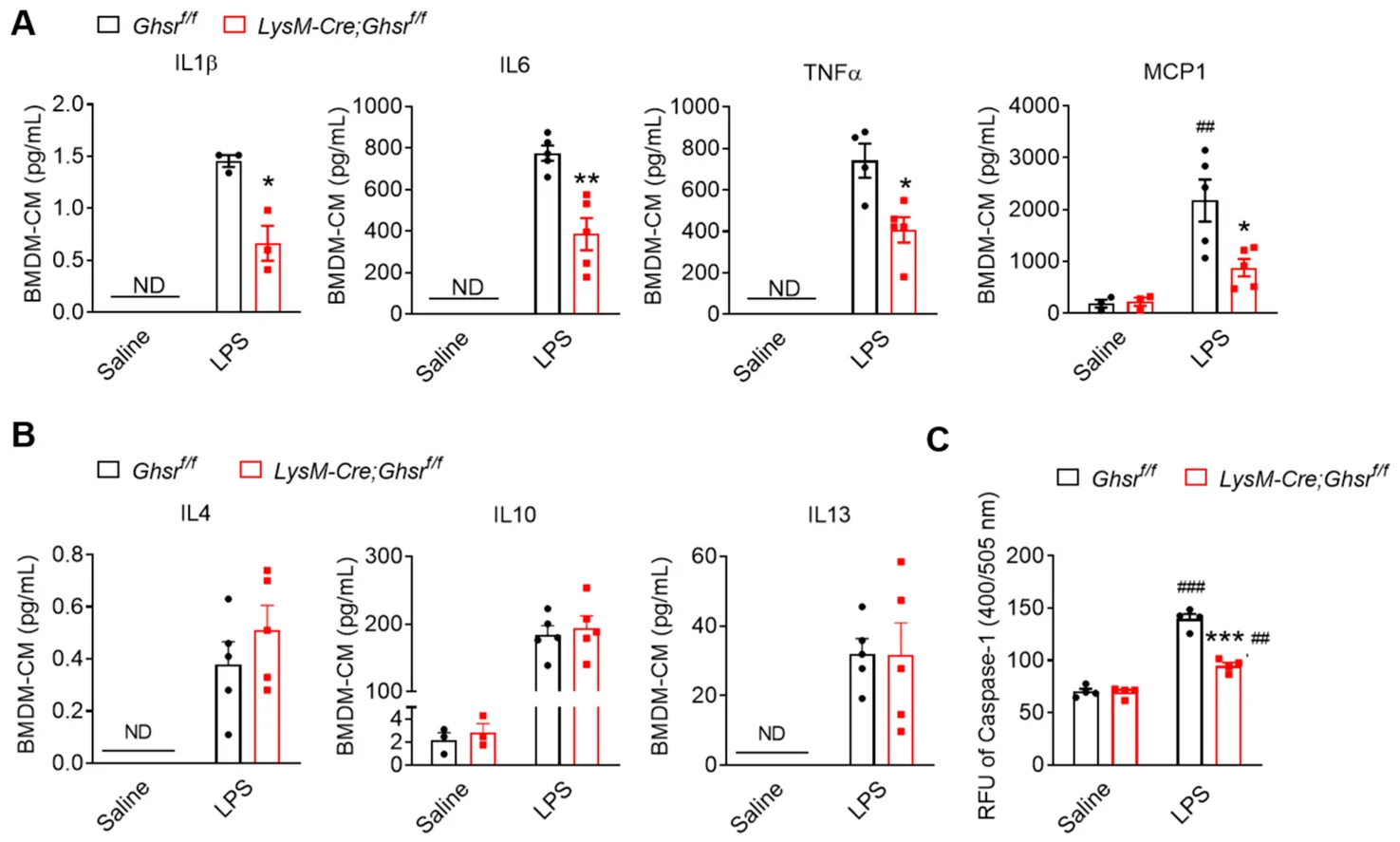
Myeloid-specific *Ghsr* deficiency attenuates LPS-induced pro-inflammatory cytokine secretion and caspase-1 activity in BMDMs. BMDMs from control (*Ghsr^f/f^*) and *LysM-Cre;Ghsr^f/f^* mice were treated with 100 ng/mL LPS. (**A**,**B**) pro-(A) and anti-(B) inflammatory cytokine/chemokine levels in BMDM-conditioned media (CM) 24 h after LPS treatment (n = 3–5/group). (**C**) Caspase-1 activity in BMDMs 4 h after LPS treatment. Data are presented as mean ± SEM. * *p* < 0.05, ** *p* < 0.01, *** *p* < 0.001, *LysM-Cre;Ghsr^f/f^* vs. *Ghsr^f/f^*; ## *p* < 0.01, ### *p* < 0.001, LPS vs. saline; ND, not detected.

To elucidate the direct effect of GHSR in macrophages, we generated BMDMs from control and *LysM-Cre;Ghsr^f/f^* mice and treated with LPS *in vitro* to induce an inflammatory state. Macrophage *Ghsr* deficiency significantly reduced LPS-induced pro-inflammatory cytokine secretion in BMDMs, including IL1β, IL6, TNFα, and MCP1 by 54.4%, 50.2%, 45.1%, and 55.0%, respectively (Figure 3A). In contrast, anti-inflammatory cytokine secretion from BMDMs was not affected (Figure 3B).

Caspase-1 is a key regulator of inflammasome complex sensing stress signals, and the activation of caspase-1 is required for cleavage and maturation of cytokine pro-forms for extracellular secretion [78]. While LPS treatment significantly increased activation of caspase-1 in control BMDMs as expected (Figure 3C), interestingly, *Ghsr*-deficient BMDMs exhibited significantly reduced LPS-stimulated caspase-1 activity (Figure 3C). These data indicate that GHSR modulates caspase-1 activation, contributing to macrophage pro-inflammatory activation under LPS-stimulated conditions.

### 3.4. Myeloid-Specific Ghsr Deletion Attenuates LPS-Induced Inflammatory Responses in Liver Tissue and Hepatocytes

**Figure 4:**
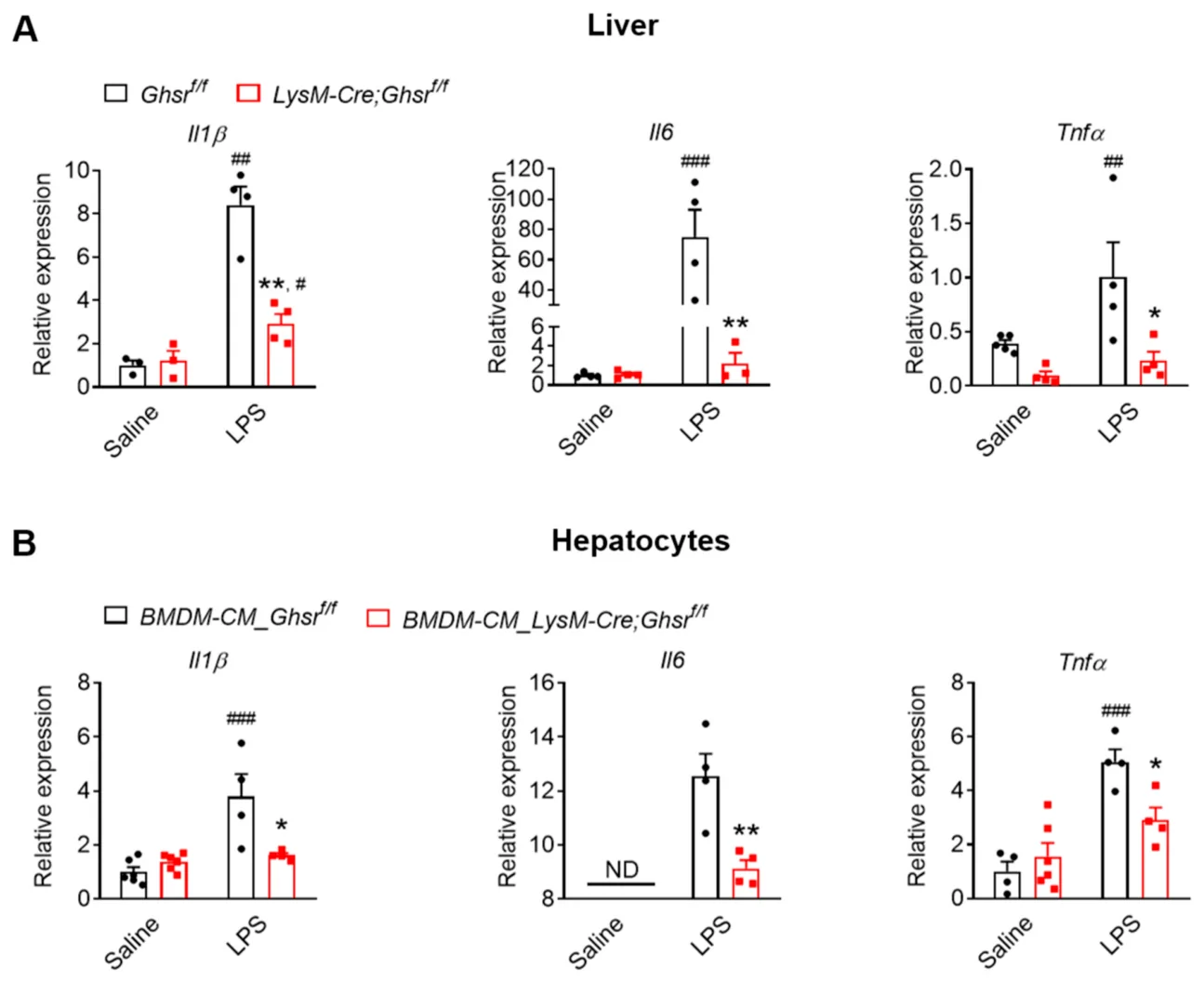
Myeloid-specific *Ghsr* deficiency reduces pro-inflammation-related gene expression in liver tissue and hepatocytes. (**A**) Gene expression of pro-inflammation-related genes in the liver 24 h following LPS *i.p.* injection, n = 3–4 mice/group. (**B**) Gene expression of pro-inflammation-related genes in control primary hepatocytes incubated with CM from LPS-treated BMDMs from *LysM-Cre;Ghsr^f/f^* and *Ghsr^f/f^* mice. 100 ng/mL LPS was used, CM was collected after 24 h, n = 3–6 biological replicates/group. Data are presented as mean ± SEM. * *p* < 0.05, ** *p* < 0.01, *LysM-Cre;Ghsr^f/f^* vs. *Ghsr^f/f^*; # *p* < 0.05, ## *p* < 0.01, and ### *p* < 0.001, LPS vs. saline; ND, not detected.

Among metabolic and immune-regulatory organs, the liver is particularly vulnerable to bacterial infection during septic shock [79,80]. High dose LPS-induced liver injury in rodents has been employed as a model to study liver failure or endotoxin-induced mortality [80,81]. We previously demonstrated that myeloid-specific *Ghsr* deficiency directly attenuates pro-inflammatory cytokine secretion [46,82]; here, we examined the effect of macrophage GHSR on the liver under endotoxemia. To investigate the effect of macrophage *Ghsr* deficiency on liver injury in LPS-induced septic endotoxemia, gene expression of pro-inflammatory cytokines in the liver of LPS-treated *LysM-Cre;Ghsr^f/f^* and control mice was examined. As expected, the expression of pro-inflammatory cytokine genes such as *Il1β*, *IL6*, and *Tnfα* was significantly increased in LPS-treated control mice (Figure 4A). Intriguingly, myeloid-specific *Ghsr*-deficient mice showed significantly reduced expression of pro-inflammatory cytokines *Il1β*, *Il6*, and *Tnfα* by 65.3%, 97.1%, and 76.9%, respectively (Figure 4A).

To further evaluate whether BMDM conditioned media (CM) of *Ghsr*-deficient BMDMs differentially modulate hepatocyte inflammatory responses, we investigated the effects of CM from LPS-treated control and *Ghsr*-deficient BMDMs on primary hepatocytes. We generated BMDMs from control and *LysM-Cre;Ghsr^f/f^* mice and isolated hepatocytes from wild-type mice. To mimic an endotoxemia condition, BMDMs were treated with LPS *ex vivo.* Primary hepatocytes from control mice were incubated with CM from ex vivo LPS-treated BMDMs from control or *LysM-Cre;Ghsr^f/f^* mice. As expected, the CM from control BMDMs significantly increased the expression of *Il1β*, *IL6*, and *Tnfα* genes (Figure 4B). Importantly, hepatocytes cultured with CM from LPS-treated *Ghsr*-deficient BMDMs exhibited significantly reduced expression of the pro-inflammatory cytokines *Il1β*, *IL6*, and *Tnfα*, with levels reduced by 57.4%, 27.2%, and 42.9%, respectively, compared to hepatocytes cultured with CM from control BMDMs (Figure 4B). Collectively, these results suggest that CM from LPS-treated *Ghsr*-deficient BMDMs is associated with reduced inflammatory gene induction in primary hepatocytes compared with CM from LPS-treated control BMDMs. These findings support a genotype-dependent CM-associated effect of macrophage *Ghsr* deficiency on hepatocyte inflammatory responses.

### 3.5. Ghsr Deficiency Attenuates LPS-Induced Mitochondrial Dysfunction in BMDMs

**Figure 5:**
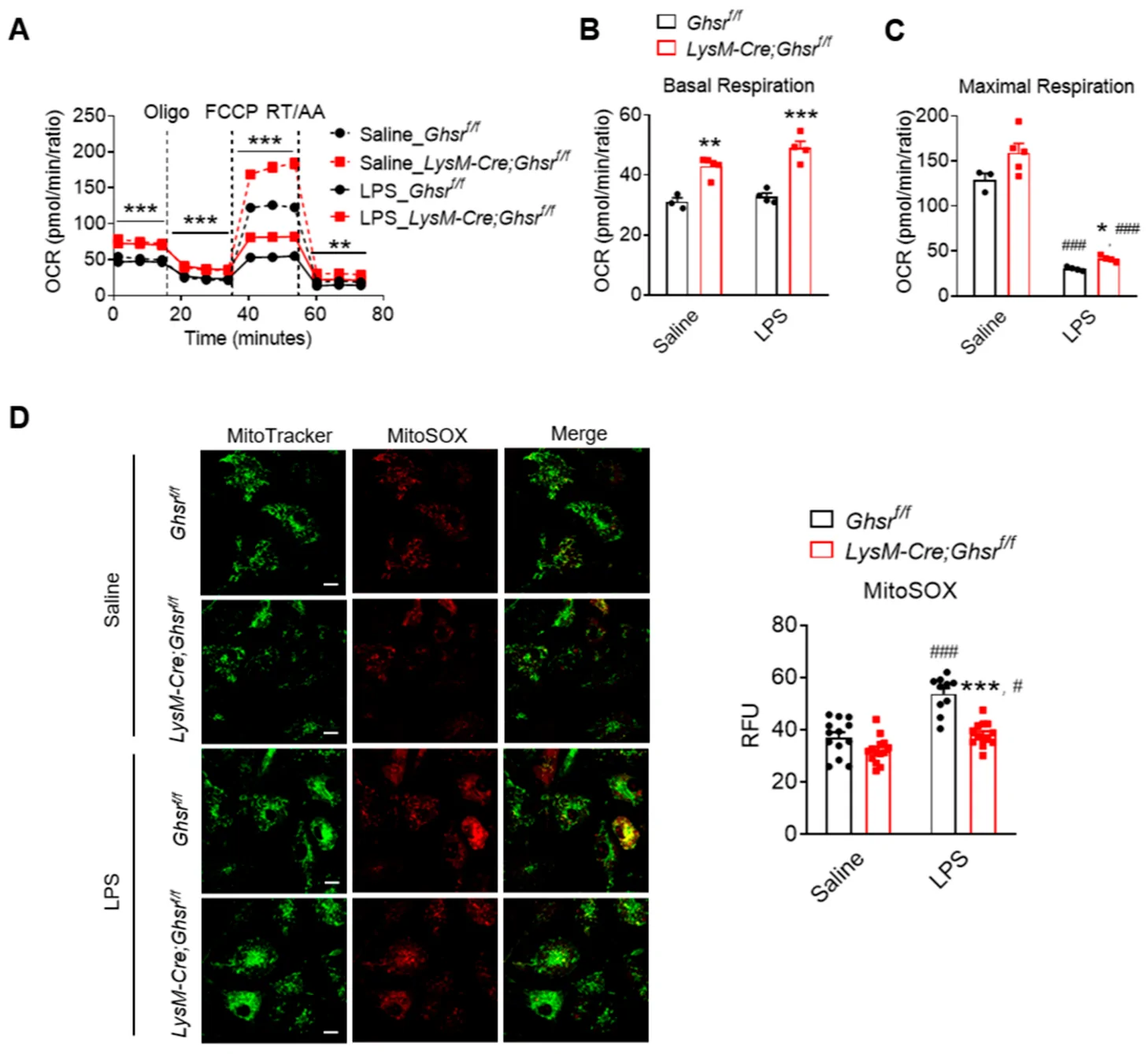
Myeloid-specific *Ghsr* deficiency improves mitochondrial oxidative phosphorylation and decreases mitochondrial superoxide in BMDMs. BMDMs were treated with 100 ng/mL LPS. (**A**) The mitochondrial oxygen consumption rate (OCR) of BMDMs was examined using the Mito Stress Kit from Seahorse Bioscience. OCR indicates the mitochondrial bioenergetic rate after 4 h of LPS treatment in BMDMs from control (*Ghsr^f/f^*) and *LysM-Cre;Ghsr^f/f^* (n = 3–5/group). Data are representative of three independent experiments, with at least three replicates per experiment. (**B**,**C**) Basal (**B**) and maximal (**C**) respiration were calculated based on the OCR profile (n = 3–5/group). Data were from three experiments, each with at least four replicates per experiment. (**D**) Mitochondrial superoxide was assessed in BMDMs from control and *LysM-Cre;Ghsr^f/f^* mice. The cultured BMDMs were treated with 100 ng/mL LPS for 60 min before staining with MitoTracker Green and MitoSOX Red, and the colocalized signals are shown in yellow in the merged images. Scale bar = 10 μm. Data were derived from at least 20 images. Data are presented as mean ± SEM. * *p* < 0.05, ** *p* < 0.01, and *** *p* < 0.001, *LysM-Cre;Ghsr^f/f^* vs. *Ghsr^f/f^*; # *p* < 0.05, ### *p* < 0.001, LPS vs. saline.

Given that mitochondrial function plays an important role in macrophage polarization, we further investigated the effects of macrophage *Ghsr* deficiency on mitochondrial function following LPS treatment. We assessed mitochondrial stress using a Seahorse Biosciences extracellular flux analyzer and found that BMDMs from *LysM-Cre;Ghsr^f/f^* mice exhibited a significantly elevated oxygen consumption rate compared to controls under both saline and LPS treatments (Figure 5A). Under LPS stimulation, *Ghsr*-deficient BMDMs exhibited significantly increased basal and maximal respiration rates compared to control BMDMs by 49.0% and 37.4%, respectively (Figure 5B,C). Moreover, we observed a significant increase in mtROS generation in control BMDMs. Remarkably, the LPS-induced increase in mtROS was attenuated in *Ghsr*-deficient BMDMs (Figure 5D). These results suggest that *Ghsr* deficiency alleviates LPS-induced mtROS production in macrophages and mitigates mitochondrial dysfunction of macrophages under an inflammatory state. Altogether, our data suggest that myeloid-specific *Ghsr* deficiency protects against LPS-induced experimental endotoxemia and endotoxemia-associated inflammatory injury, in part by attenuating pro-inflammatory activation of macrophages, which ultimately mitigates severe liver damage and organ failure.

## 4. Discussion

**Figure 6:**
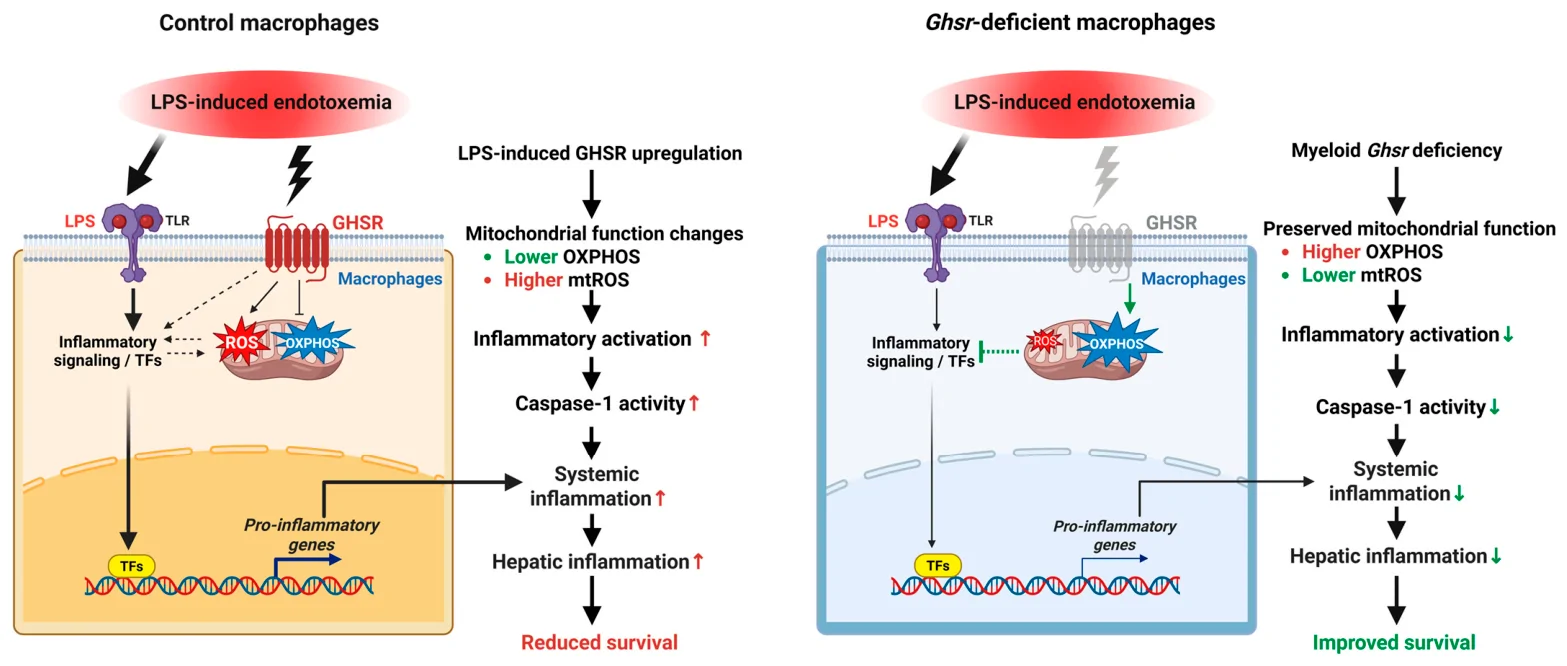
Proposed model of macrophage GHSR in LPS-induced endotoxemia. In control macrophages, LPS challenge is associated with increased GHSR expression, reduced OXPHOS, increased mitochondrial ROS production, enhanced inflammatory activation by promoting inflammatory signaling/transcription factors (TFs), and increased caspase-1 activity, collectively contributing to systemic and hepatic inflammation and reduced survival in endotoxemia. In contrast, myeloid *Ghsr* deficiency is associated with enhanced mitochondrial respiration, reduced mtROS production, attenuated inflammatory activation, and reduced caspase-1 activity, contributing to reduced systemic and hepatic inflammation and improved survival following LPS-induced endotoxemia.

Endotoxemia, the presence of LPS in the bloodstream, triggers systemic inflammation and occurs during severe infections like sepsis, which is a life-threatening disease with extreme mortality. The pathological signatures include mitochondrial dysfunction, oxidative stress, and impaired immune responses, which lead to cytokine “storm” and organ failure [83,84]. In the current study, we found that GHSR protein levels in macrophages were significantly increased by 6 h of LPS treatment, and myeloid-specific *Ghsr* deficiency mitigated LPS-induced systemic and tissue inflammation by suppressing inflammatory responses in macrophages. Our findings are supported by several lines of compelling *in vivo* and *in vitro* evidence: (1) In response to LPS treatment, myeloid-specific *Ghsr*-deficient mice exhibited lower levels of systemic (serum) and tissue (liver) inflammation under LPS-induced endotoxemia. Myeloid-specific *Ghsr*-deficient mice displayed an improved survival following LPS-induced endotoxemia, indicating that *Ghsr* deficiency may have a protective effect against endotoxemia-induced mortality; (2) In response to LPS stimulation *in vitro*, *Ghsr*-deficient BMDMs showed reduced pro-inflammatory cytokine secretion and suppressed caspase-1 activity, suggesting attenuated inflammasome-associated inflammatory responses. Moreover, *Ghsr* deficiency enhanced mitochondrial OXPHOS activity, consistent with a metabolic profile generally associated with reduced inflammatory activation. Consistently, *Ghsr* deficiency resulted in reduced mtROS levels. Together, these findings strongly suggest that myeloid GHSR deficiency alleviates LPS-induced mitochondrial dysfunction. These novel findings reveal that macrophage GHSR plays an important role in the regulation of acute inflammatory responses and contributes to endotoxemia-associated immune activation (Figure 6). Thus, macrophage GHSR has the potential to be a therapeutic target for the treatment of endotoxemia.

Our results show that myeloid-specific *Ghsr* deficiency effectively suppresses pro-inflammatory cytokines in plasma under LPS-induced endotoxemia, indicating that macrophage *Ghsr* deficiency attenuates systemic pro-inflammatory cytokine responses during endotoxemia. We recently reported that *Ghsr*-deficiency reduces nuclear translocation and phosphorylation of NF-κB in macrophages [46,54]. NF-κB is a key signaling pathway in control of inflammasome activation. The activated inflammasome triggers caspase-1 to process the precursor forms of its substrates such as pro-IL1β into the biologically active form IL1β [33,85]. Here, we found that myeloid-specific *Ghsr* deficiency decreased caspase-1 activity and cytokine expression in macrophages in response to LPS exposure. This result suggests that the attenuated inflammatory response of *Ghsr*-deficient macrophages under endotoxemia is at least partly due to reduced NF-κB nuclear translocation and IL-1β processing.

It has been previously demonstrated that LPS-induced liver damage is associated with inflammatory mediators such as IL1β and TNFα [86,87,88]. We recently reported that myeloid-specific *Ghsr* ablation attenuates CCl4-induced liver fibrosis and inflammation, evidenced by reduced hepatic monocyte-derived macrophages and suppressed pro-inflammatory responses [82]. Similar to these studies, here we observed that the liver inflammatory phenotype was markedly attenuated in myeloid-specific *Ghsr*-deficient mice under LPS-induced endotoxemia. Furthermore, in the co-culture system, we found that hepatocytes exposed to CM from LPS-treated *Ghsr*-deficient BMDMs exhibited significantly reduced expression of pro-inflammatory cytokines compared with those treated with CM from LPS-treated control BMDMs. Together, these data demonstrate that myeloid-specific *Ghsr* deficiency attenuates immune activation in the circulation, liver tissue, and *ex vivo* culture under an endotoxemia state, supporting a critical role for macrophage GHSR signaling in the hepatic regulation of inflammatory responses in LPS-induced endotoxemia.

Several lines of evidence show that ROS play a leading role in septic shock and organ failure in sepsis patients due to their effects on mitochondrial impairment, inflammation activation, and cell apoptosis [89,90,91]. Previous studies have shown that mitochondria regulate inflammatory signaling in innate immune cells through the generation of mtROS, and that excessive mitochondrial oxidative stress can amplify inflammatory responses and contribute to tissue injury [29,30]. Indeed, our findings show that GHSR deficiency alleviated mitochondrial dysfunction in macrophages under septic endotoxemia. Specifically, under endotoxemic LPS challenge, *Ghsr* deficiency suppressed the generation of mtROS and attenuated mitochondrial impairment in macrophages. Our data show that macrophage GHSR deficiency reduces mtROS production, potentially through enhanced mitochondrial antioxidant scavenging capacity and/or decreased electron leak from the respiratory chain (e.g., improved coupling/organization of ETC complexes) [92]. Under oxidative stress, macrophages undergo extensive changes in glycolysis and oxidative phosphorylation, resulting in inflammatory polarization and elevated pro-inflammatory cytokine production [93]. The activation of macrophages by endotoxins, such as LPS, results in metabolic reprogramming of macrophages exhibiting a glycolytic burst and suppressed OXPHOS [94]. We previously demonstrated that *Ghsr*-deficient macrophages exhibit reduced LPS-induced glycolysis in macrophages [46]. Here, we show that *Ghsr*-deficient BMDMs exhibited increased OCR at basal and maximal respiration under vehicle treatment, indicating enhanced mitochondrial OXPHOS in the basal state. This observation is consistent with our previous study, in which GHSR was shown to enhance basal respiration under BSA and palmitic acid (PA) treatments [46]. Together, these findings suggest that GHSR contributes to the intrinsic metabolic programming of macrophages under basal conditions, potentially by modulating mitochondrial function, substrate utilization, and/or energy homeostasis. Importantly, we observed that *Ghsr*-deficient macrophages exhibited higher maximal OCR compared with control macrophages under LPS stimulation, indicating improved mitochondrial OXPHOS under LPS-induced inflammatory stress. Taken together, the data suggest that macrophage GHSR is involved in the shift of metabolic pathways from mitochondrial OXPHOS to glycolysis in response to LPS. This metabolic reprogramming contributes to macrophage M1 pro-inflammatory polarization. Further studies dissecting the underlying mechanisms, including the specific signaling pathway(s) through which GHSR regulates mtROS and OXPHOS, will help to better understand the precise roles of GHSR in mitochondrial reprogramming of macrophages.

Several limitations of this study should be acknowledged. First, our findings were based on an acute LPS-induced endotoxemia model, which reproduces systemic inflammatory responses to endotoxin exposure but does not fully recapitulate the complexity of clinical sepsis, such as polymicrobial infection, pathogen burden, and dynamic host-pathogen interactions. Therefore, the protective effects of myeloid-specific *Ghsr* deficiency observed in this model should not be directly extrapolated to sepsis. Second, although our data demonstrate associations between *Ghsr* deficiency, reduced inflammatory responses, decreased mtROS production, and enhanced mitochondrial OXPHOS, they do not necessarily support a direct effect of macrophage GHSR in mitochondrial reprogramming. Therefore, further mechanistic studies will be required to determine how GHSR regulates mitochondrial function and inflammatory signaling in macrophages. In addition, the co-culture study of BMDM-CM cannot distinguish the paracrine effects of BMDM-derived factors from direct effects of residual LPS on hepatocytes. Although our results provide evidence that BMDM-derived soluble factors in GHSR deficiency modulate hepatocyte inflammatory gene expression, further studies with additional conditions, such as LPS-containing medium alone, are needed to definitively evaluate the specific paracrine effects of macrophage-derived factors. Finally, our experiments primarily focused on macrophages and the liver; the effects of GHSR signaling in other myeloid populations and other organs altered in endotoxemia remain to be determined. Future studies with more clinically relevant sepsis models and in-depth mechanistic approaches would be necessary to further define the role of macrophage GHSR in systemic inflammation and mitochondrial regulation.

## 5. Conclusions

In summary, our study suggests that myeloid GHSR has an important role in LPS-induced endotoxemia, and GHSR deficiency in myeloid cells improves survival in acute endotoxemia. Our data demonstrate that myeloid *Ghsr* deficiency is associated with improved mitochondrial function and reprograms inflammatory activation of macrophages, which in turn attenuates systemic cytokine storm and tissue damage. The findings suggest that GHSR has the potential to serve as an immunotherapeutic target for endotoxemia.

## Data Availability

The raw data supporting the conclusions of this article will be made available by the authors upon request.
